# Outcomes of Malperfusion Post–Aortic Dissection Repair With the AMDS Hybrid Prosthesis: The DARTS Trial Post Hoc Analysis

**DOI:** 10.1016/j.jacadv.2026.102991

**Published:** 2026-07-09

**Authors:** Ryaan EL-Andari, Sabin J. Bozso, Jeevan Nagendran, Michael W.A. Chu, Maral Ouzounian, Bob Kiaii, Ismail El-Hamamsy, Jessica Forcillo, Jorg Kempfert, Michael C. Moon

**Affiliations:** a Division of Cardiac Surgery, University of Alberta, Edmonton, Canada; b Division of Cardiovascular Surgery, Sanger Heart and Vascular Institute, Charlotte, North Carolina, USA; c Division of Cardiac Surgery, Department of Surgery, Western University, London, Canada; d Division of Cardiac Surgery, University of Toronto, Toronto, Canada; e Division of Cardiac Surgery, Libin Cardiovascular Institute, University of Calgary, Calgary, Canada; f Department of Cardiovascular Surgery, Icahn School of Medicine at Mount Sinai, New York, New York, USA; g Division of Cardiac Surgery, Centre Hospitalier de L Université de Montréal (CHUM), Montreal, Canada; h Department of Cardiothoracic and Vascular Surgery, Deutsches Herzzentrum der Charité, Berlin, Germany

**Keywords:** AMDS, aorta

**What is the clinical question being addressed?**

Are there differences in all-cause mortality and major morbidity in patients with and without malperfusion undergoing aortic dissection repair with the AMDS Hybrid Prosthesis?

**What is the main finding?**

Patients with malperfusion experience similar mortality to patients without malperfusion following aortic dissection repair with the AMDS Hybrid Prosthesis.

The DARTS (Dissected Aorta Repair Through Stent) Implantation trial demonstrated the safety and efficacy of the AMDS Hybrid Prosthesis (Artivion).^1^^,^^2^ A key finding of the DARTS data was postoperative resolution of malperfusion. This has been reflected in the PERSEVERE trial which has exclusively enrolled patients with malperfusion.^3^

In previous cohorts, preoperative malperfusion has been associated with operative mortality rates of 20% to 40%.^4^^,^^5^ A direct comparison between patients with and without malperfusion in those who received the AMDS has not been performed. Herein, we perform a post hoc analysis of the DARTS trial comparing the outcomes of patients with and without malperfusion over a 5-year follow-up period.

The DARTS trial is a prospective, multicenter, single-arm trial with methods previously described.^1^ The inclusion criteria were patients 18 to 80 years of age diagnosed with acute DeBakey type I (ADTI) or intramural hematoma. The exclusion criteria were extreme hemodynamic compromise, aortic arch aneurysm, or connective tissue disease. Ethics approval was obtained by the participating sites. Approval at the principal investigator's site for Pro00066039 was obtained on January 11, 2019, with written informed consent obtained following ADTI repair and waiver of consent where patients did not survive the perioperative period.

Patients were grouped based on the presence of preoperative clinical or radiographic malperfusion. The primary outcome was all-cause mortality. Secondary outcomes included major morbidity. 5-year survival was compared based on malperfusion using the Log-rank test. Fisher exact test was used to compare differences in binary variables whereas unpaired 2-tailed t-tests were used to compare differences in continuous variables. Cox proportional hazard regression model was fitted to evaluate the effect size of malperfusion on the long-term primary and secondary outcomes reported as HRs with 95% CIs. Statistical analyses were performed via GraphPad Prism (GraphPad).

The DARTS trial cohort consisted of forty-seven patients, 1 of which was excluded for off-label use. The trial enrolled patients undergoing ADTI repair from March 2017 to January 2019. Five-year follow-ups were completed by June 2024. A total of 25 patients had malperfusion (15 radiographic and 10 clinical) and 21 did not. Baseline demographics of patients with and without malperfusion were similar.

There was no significant difference in operative mortality with 19.1% mortality for patients without malperfusion and 12.0% for patients with malperfusion (*P* = 0.52). Five-year survival was 71.1% in patients without malperfusion and 74.3% in patients with malperfusion (*P* = 0.68) (Figure 1). In the time-to-event analysis, malperfusion was not associated with a significantly accelerated hazard of mortality over the 5-year period (HR: 0.79; 95% CI: 0.3-2.5).

**Figure 1:**
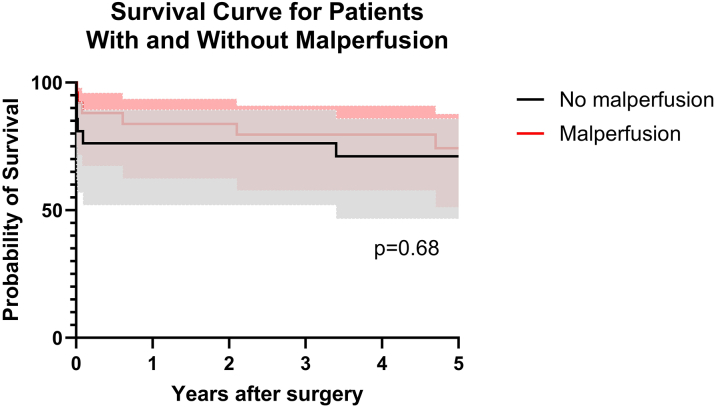
**Kaplan-Meier Curve Comparing Survival for Patients With and Without Malperfusion** The log-rank test was used to compare survival between groups (*P* = 0.68). Shaded areas represent 95% CIs.

New stroke occurred in 4.8% of patients without malperfusion and 36.0% of patients with malperfusion (*P* = 0.01). In the time-to-event analysis, malperfusion was associated with a significantly accelerated hazard of stroke over the 5-year period; however, due to limited occurrences, the CI was notably wide (HR: 9.0; 95% CI: 1.7-165.3). Other outcomes such as major bleeding (14.3% without malperfusion vs 8.0% with malperfusion; *P* = 0.65), acute renal failure (14.3% vs 16.0%; *P* = 0.99), and delirium (4.7% vs 12.0%; *P* = 0.61) were not significantly different.

Patients presenting with ADTI and preoperative malperfusion are known to have higher rates of morbidity and mortality.^4^^,^^5^ Previous analyses of the DARTS trial found high rates of resolution of malperfusion with the AMDS, although comparative outcomes based on malperfusion following repair with the AMDS have not been performed. In a study of the Society of Thoracic Surgeons database with 9,958 patients, Goel et al identified rates of operative mortality for patients with aortic dissection without malperfusion to be 13.6%, this is lower than the 19% 30 day mortality for the nonmalperfused patients in the DARTS study but numerically higher than the 12% 30 day mortality for the malperfused patients in the DARTS cohort. In the study by Goel et al^4^, operative mortality for patients with malperfusion was 26.8% with specific types of malperfusion resulting in even higher mortality (41.2% for coronary, 34.3% for mesenteric, 34.7% for spinal malperfusion).

This study found that patients experienced similar 5-year outcomes post-ADTI repair irrespective of malperfusion. Rates of new stroke were higher in the malperfused group, which may have been related to preoperative cerebral malperfusion, which was not present in the nonmalperfusion group. This post hoc analysis was not in the initial trial protocol, potentially resulting in the comparison being underpowered and, notably, there was no control group that did not receive the AMDS. However, lower-than-expected mortality was identified for patients with malperfusion, comparable to patients without malperfusion. This suggests that the AMDS hybrid prosthesis is effective in mitigating the negative impact of malperfusion on postoperative outcomes, resulting in similar outcomes irrespective of the presence of malperfusion, in contrast to the previous literature.

## Funding support and author disclosures

The DARTS Trial is sponsored by Artivion. Dr Moon has received consulting fees from Artivion. Dr Chu has received speaker honoraria from Medtronic, Edwards Lifesciences, Terumo Aortic, and Artivion. All other authors have reported that they have no relationships relevant to the contents of this paper to disclose.
